# Exploratory analysis of exhaled volatile organic compounds for binary discrimination between lung cancer, pneumonia, and healthy controls using machine learning

**DOI:** 10.3389/fmed.2026.1741424

**Published:** 2026-02-23

**Authors:** Jing Wang, Haitian Li, Jianshen Yue, Yamei Song, Ning Wang, Wei Guo, Zhigang Cai

**Affiliations:** 1 The First Department of Pulmonary and Critical Care Medicine, The Second Hospital of Hebei Medical University, Shijiazhuang, China; 2 Department of Infectious Disease, The People’s Hospital of Hengshui, Hengshui, China; 3 The First Department of Pulmonary and Critical Care Medicine, The People’s Hospital of Cangzhou, Cangzhou, China; 4 Department of Pulmonary and Critical Care Medicine, The People’s Hospital of Hengshui, Hengshui, China; 5 Hebei Key Laboratory of Respiratory Critical Care Medicine, Shijiazhuang, China; 6 Hebei Institute of Respiratory Diseases, Shijiazhuang, China

**Keywords:** exhaled breath analysis, exploratory study, lung cancer, machine learning, pneumonia, volatile organic compounds

## Abstract

**Background:**

Lung cancer remains a major cause of cancer-related mortality worldwide, while pneumonia is one of the most prevalent infectious diseases, with acute pneumonia being highly common globally. Despite continuous advancements in diagnostic technology and the successive launch of new anti-infective drugs, the incidence and mortality rates of pneumonia remain high. Exhaled breath volatile organic compounds (VOCs) have been proposed as non-invasive indicators of disease-related metabolic and pathophysiological alterations. Lung cancer and pneumonia often present with similar nodules or consolidation shadows on chest imaging, leading to frequent diagnostic overlap and delays. This uncertainty can cause lung cancer patients to miss the optimal treatment window or result in unnecessary invasive examinations for pneumonia patients. The current gold standard for definitive diagnosis relies on invasive methods, but it has drawbacks such as operational risks, patient discomfort, radiation exposure, and high costs. Therefore, this study was designed as an exploratory, proof-of-concept investigation to examine whether VOC profiles exhibit distinguishable patterns between lung cancer, pneumonia, and healthy individuals using pairwise binary analytical approaches.

**Methods:**

Exhaled breath samples were collected from participants with lung cancer (*N* = 180), pneumonia (*N* = 228), and healthy controls (*N* = 180). Samples were analyzed using a micro gas chromatography system coupled with a mass spectrometry detector (micro-GC–MSD). Univariate statistical analyses were performed to screen for VOCs showing differential abundance between groups. Multivariate analyses were subsequently conducted using five machine learning algorithms to evaluate the discriminative performance of VOC-based models in pairwise binary comparisons between lung cancer and healthy controls, pneumonia and healthy controls, and lung cancer and pneumonia.

**Results:**

Multiple VOCs demonstrated statistically significant differences between groups, although substantial overlap in distributions was observed. Compared with healthy controls, three VOCs (heptane, propane, 1-(methylthio)-, and styrene) showed lower levels and two VOCs (2-hexanone, 6-hydroxy- and o-xylene) showed higher levels in the lung cancer group. In the pneumonia group, six VOCs (1,4-pentadiene, toluene, butyl acetate, p-xylene, D-limonene, and isobutyl nonyl carbonate) were elevated, while one VOC (heptane, 2,2,4,6,6-pentamethyl-) was reduced compared with healthy controls. In pairwise comparisons between lung cancer and pneumonia, seven VOCs showed lower concentrations in the lung cancer group. With area under the receiver operating characteristic curve (AUC) values of 0.980 for lung cancer versus healthy controls, 0.956 for pneumonia versus healthy controls, and 0.983 for lung cancer versus pneumonia.

**Conclusion:**

This exploratory study demonstrates that exhaled breath VOC profiles, analyzed via machine learning, yield statistically distinguishable signals in pairwise comparisons between lung cancer, pneumonia, and healthy individuals. These results provide preliminary evidence that breath analysis could address the critical clinical challenge of differentiating radiographically similar conditions non-invasively. The presented methodology and dataset establish a foundational framework for characterizing disease-specific metabolic signatures. However, the findings remain hypothesis-generating. Definitive evaluation of clinical utility necessitates subsequent studies employing multiclass modeling, validation in independent and prospective cohorts, and direct assessment of diagnostic impact in real-world triage scenarios.

## Background

Lung cancer remains the leading cause of cancer-related mortality worldwide and is among the most frequently diagnosed malignant tumors. According to the International Agency for Research on Cancer (IARC), approximately 20 million new cancer cases and 9.7 million cancer-related deaths were reported globally in 2022, with lung cancer accounting for about 2.5 million new cases (12.4%) and 1.8 million deaths (18.7%), representing the highest incidence and mortality among all malignancies (1). East Asia bears a particularly high disease burden, contributing nearly half of global lung cancer cases, with China alone accounting for more than 40%. Despite advances in imaging, pathology, and systemic therapies, overall survival remains poor, largely because most cases are identified at advanced stages. These epidemiological trends have motivated continued efforts to improve strategies for earlier disease characterization and risk stratification.

In clinical practice, lung cancer assessment relies primarily on low-dose computed tomography (LDCT), bronchoscopy, and tissue biopsy. Although LDCT has been widely adopted in screening programs, it is associated with a high false-positive rate, reported to be approximately 27%, leading to unnecessary follow-up procedures and patient anxiety. Invasive approaches such as bronchoscopy and biopsy are limited by procedural risks and reduced sensitivity for small or peripherally located lesions. Moreover, early clinical manifestations of lung cancer, including cough, dyspnea, and chest discomfort, are non-specific and frequently overlap with those of pulmonary infections. Pneumonia itself remains highly prevalent and is commonly evaluated using chest imaging, which may be constrained by radiation exposure, contrast-related risks, and limited suitability for certain populations. Notably, pneumonic-type lung cancer can present radiographic features that closely resemble pneumonia, increasing the likelihood of diagnostic uncertainty and delayed clinical decision-making.

Against this background, increasing attention has been directed toward non-invasive approaches capable of capturing disease-associated biological information beyond conventional imaging. Volatile organic compounds (VOCs) present in exhaled breath arise from a combination of endogenous metabolic processes and exogenous exposures. Endogenous VOCs are generated through cellular metabolism and transported to the alveoli via blood–gas exchange, whereas exogenous VOCs may originate from diet, environmental exposure, occupational factors, or microbial activity within the oral and gastrointestinal microbiota (2). Because exhaled breath collection is non-invasive, repeatable, and associated with minimal patient burden, breath-based VOC analysis has emerged as a valuable research tool for investigating metabolic alterations linked to respiratory diseases.

Since the pioneering work by Gordon et al. (3), numerous studies have explored breath VOC profiles in lung cancer and other respiratory conditions using analytical platforms such as gas chromatography–mass spectrometry (GC–MS), proton transfer reaction–mass spectrometry (PTR–MS), and electronic nose (eNose) technologies (4–6). Reported findings suggest that VOC patterns may differ between disease states, including chronic obstructive pulmonary disease, asthma, lung cancer, and COVID-19 (7). However, existing studies vary substantially in analytical techniques, feature selection strategies, and statistical modeling approaches, leading to inconsistent results. Importantly, many investigations rely on pairwise binary comparisons and often infer diagnostic potential without implementing or validating multiclass classification frameworks. As a result, the clinical interpretability and generalizability of VOC-based findings remain uncertain.

In this context, the present study was designed as an exploratory, proof-of-concept investigation to examine whether exhaled breath VOC profiles exhibit statistically distinguishable patterns between individuals with lung cancer, pneumonia, and healthy controls. Using micro-GC–MS analysis combined with univariate statistics and machine learning–based binary classification models, we aimed to characterize VOC differences and assess their discriminative performance in pairwise comparisons. This work seeks to contribute to a better understanding of disease-associated VOC signatures.

## Materials and methods

### Participants

From May 15, 2023 to May 15, 2025, a total of 588 participants were enrolled at Hengshui People’s Hospital, including patients with lung cancer (*n* = 180), patients with pneumonia (*n* = 228), and healthy controls (*n* = 180). Patients were recruited from clinical departments of the hospital, while healthy controls were recruited from individuals undergoing routine physical examinations at the hospital’s health screening center. Recruitment and breath sample collection were conducted at Hengshui People’s Hospital in accordance with protocols approved by the institutional Ethics Committee. All participants provided written informed consent and completed a standardized questionnaire.

All participants underwent pulmonary function testing, and only individuals with normal lung function were included to exclude chronic obstructive pulmonary disease and other conditions associated with impaired ventilation.

Patients with lung cancer were eligible if they were aged 18–80 years, had imaging findings highly suggestive of malignancy, and had a histopathological diagnosis of non-small cell lung cancer. Exclusion criteria included confirmation of small cell lung cancer, severe pulmonary dysfunction preventing breath sampling, a history of other primary malignancies, or severe systemic diseases.

Patients with pneumonia were eligible if they were aged ≥ 18 years and had a diagnosis of pulmonary infection based on clinical presentation, laboratory findings, imaging, and microbiological evidence, with no prior treatment initiated at the time of breath sampling. Exclusion criteria included pulmonary lesions attributable to autoimmune or vascular diseases (e.g., sarcoidosis, Wegener’s granulomatosis), heart failure, lung cancer, severe systemic comorbidities, multisystem infections, or critical illness precluding participation.

Healthy controls were eligible if they were aged 18–80 years, had no abnormal findings on chest imaging, no recent respiratory infections, and no history of malignancy.

### Exhaled breath collection

Participants were instructed to avoid alcohol, caffeine, garlic, chili, smoking, and other potential VOC-interfering substances for at least 12 h prior to sampling, and to refrain from eating or drinking for 1 h before sample collection. Immediately before sampling, participants rinsed their mouths with water for 1 min to reduce oral contaminants. These pre-collection restrictions were waived for pneumonia patients requiring urgent clinical care.

Breath sampling was conducted in a controlled laboratory environment. Participants were instructed to avoid speaking or coughing during collection. Using a medical-grade disposable mask connected directly to the sampling inlet and constructed from inert polymer materials, participants inhaled normally through the nose and exhaled slowly through the mouth for a total sampling duration of 3 min. This nasal inhalation–oral exhalation protocol was adopted to reduce ambient air contamination and preferentially sample lower respiratory tract gas.

Between participants, the analytical system was flushed with nitrogen to eliminate residual compounds and prevent cross-contamination.

### Instrumentation and VOC analysis

Exhaled breath samples were analyzed using a micro gas chromatography system (CXBA-Alpha, ChromX Health Co., Ltd.) equipped with an integrated detection module. The system consisted of a μPCI chip for VOC capture and thermal desorption, a 10-m microcolumn chip for temperature-programmed chromatographic separation, and a μPID detector for real-time signal acquisition. Approximately 600 mL of exhaled breath was introduced directly into the system without the use of gas storage bags.

Water vapor was removed using a Nafion drying tube prior to VOC enrichment. VOCs were concentrated on the μPCI chip and immediately thermally desorbed into the microcolumn for separation and detection.

To enable compound identification, 20% of samples were simultaneously diverted to adsorption tubes and analyzed offline using a mass spectrometry detector (MSD). Mass spectral data were processed using MassHunter Qualitative Analysis software (version 10.0) and matched against the NIST 2017 (v2.3) mass spectral library.

### Data preprocessing and peak area quantification

Chromatographic data were processed using Python (v3.9.19). Each chromatogram underwent spike removal, baseline correction, detector overload correction, and windowed Gaussian smoothing. Peaks were detected using a second-derivative–based algorithm, with potential co-eluting clusters identified automatically. Overlapping peaks were resolved using Gaussian mixture model fitting, and the integrated peak area of each resolved compound was calculated as the quantitative measure.

Peaks were aligned across samples based on retention-time similarity to construct a VOC abundance matrix. VOCs were retained if detected in at least 80% of all samples or in at least 80% of samples within any single clinical group. Samples were excluded if fewer than 80% of retained VOCs were successfully quantified.

To correct for day-to-day instrumental variability, a daily correction factor (κ) was calculated as the mean peak area of six anchor compounds (isoprene, n-non-ane, α-pinene, n-decane, (R)-(+)-limonene, and n-undecane). All VOC peak areas were normalized using the formula: V′ = 100 × V/κ.

### Quality control

Routine quality control was performed every 3 days using standard gas mixtures containing known concentrations of n-heptane, p-xylene, and styrene stored in a Masu canister. Background air was collected for 3 min prior to QC sample analysis. Samples failing QC criteria were reanalyzed. Instrumental drift correction was performed automatically by the system and verified by an engineer.

### Feature selection

Univariate and multivariate approaches were applied for exploratory feature screening. All feature selection steps (including univariate statistical tests and VIP-based filtering) were strictly performed within the training set. Specifically, the dataset was first divided into training and test sets according to a stratified random sampling strategy, and feature selection was subsequently conducted on the training set. This process effectively avoids optimistic bias caused by prematurely using test set information. The Mann–Whitney U test was used for pairwise group comparisons, with false discovery rate (FDR) correction applied for multiple testing. VOCs with adjusted *p* < 0.05 were considered statistically significant.

Orthogonal partial least squares discriminant analysis (OPLS-DA) was used as an exploratory multivariate method. Variables with a variable importance in projection (VIP) score > 1 were considered to contribute substantially to group separation.

### Machine learning analysis

Machine learning analyses were conducted exclusively using pairwise binary classification frameworks: lung cancer vs. healthy controls, pneumonia vs. healthy controls, and lung cancer vs. pneumonia. Five algorithms were evaluated: logistic regression, support vector classification, k-nearest neighbors, random forest, and extreme gradient boosting. Multiclass classification was intentionally not pursued in order to avoid inflated performance estimates in the absence of sufficient external validation.

Data were partitioned using stratified sampling to preserve class proportions. The dataset is stratified random sampling, which divides it into a training set and an independent test set, to ensure that the proportion of each classification label is consistent in both subsets. To ensure reproducibility, we set a random seed of 42 for the entire analysis process, covering data splitting, model training, and hyperparameter optimization. Performance metrics (such as accuracy, AUC, etc.) are calculated based on the independent test set obtained from a single data split, and the final performance report is not based on repeated sampling. However, to assess the stability of the performance estimate, we further use the Bootstrap resampling method (*n* = 1000) to build 95% confidence intervals for each metric, providing more robust results.

Performance metrics included area under the ROC curve (AUC), accuracy, sensitivity, specificity, F1 score, positive predictive value, and negative predictive value. Non-parametric bootstrapping with 1,000 resamples was used to estimate 95% confidence intervals.

### Statistical analysis

Statistical analyses were performed using SPSS 26.0 and R (version 4.4.2). Normality was assessed using the Shapiro–Wilk test. Non-normally distributed variables were summarized as median (interquartile range). Categorical variables were compared using chi-square or Fisher’s exact tests as appropriate. All statistical tests were two-sided, and *p* < 0.05 was considered statistically significant.

## Results

### Participant characteristics

A total of 588 participants were included in the analysis, comprising 180 patients with lung cancer, 228 patients with pneumonia, and 180 healthy controls. Demographic and clinical characteristics of the study population are summarized in Table 1. Overall, the three groups were comparable with respect to sex distribution, age, body mass index (BMI), smoking status, and alcohol consumption.

**TABLE 1:** Demographic characteristics of the participants.

Variable	Lung cancer (*N* = 180)	Pneumonia (*N* = 288)	Healthy participants (*N* = 180)	*P*-values
Sex (male)	96 (53.3)	120 (52.6)	101 (56.1)	0.052
Age, years	53.85 ± 17.94	55.28 ± 17.66	51.06 ± 16.91	0.051
Body mass index, kg/m^2^	23.3 ± 6.3	22.8 ± 4.8	23.2 ± 4.1	0.555
**Smoking status**				
Never	95 (52.8)	125 (54.8)	96 (53.3)	0.973
Current	54 (30.0)	67 (29.4)	48 (26.7)	0.755
Quit within past year	31 (17.2)	36 (15.8)	36 (20.0)	0.536
**Alcohol use**				
Never	93 (51.7)	124 (54.4)	96 (53.3)	0.861
Occasionally	69 (38.3)	70 (30.7)	48 (26.7)	0.054
Regular	18 (10.0)	34 (14.9)	36 (20.0)	0.383

Data are (%) or mean ± SD.

Male participants accounted for 53.3% of the lung cancer group, 52.6% of the pneumonia group, and 56.1% of the healthy control group. Mean age was 53.85 ± 17.94 years in the lung cancer group, 55.28 ± 17.66 years in the pneumonia group, and 51.06 ± 16.91 years in healthy controls. Mean BMI values were similar across groups. Smoking status and alcohol consumption patterns were also comparable. With respect to smoking status, approximately half of participants in each group reported never having smoked, including 52.8% in the lung cancer group, 54.8% in the pneumonia group, and 53.3% among healthy participants. Current smokers comprised 30.0% of the lung cancer group, 29.4% of the pneumonia group, and 26.7% of the healthy participant group. Participants who had quit smoking within the past year accounted for 17.2%, 15.8%, and 20.0% of the lung cancer, pneumonia, and healthy groups, respectively. Approximately half of participants reported no alcohol use, including 51.7% of patients with lung cancer, 54.4% of patients with pneumonia, and 53.3% of healthy participants. Occasional alcohol use was reported by 38.3% of the lung cancer group, 30.7% of the pneumonia group, and 26.7% of healthy participants, whereas regular alcohol consumption was reported by 10.0%, 14.9%, and 20.0% of participants in the respective groups.

### Differential VOC profiles in pairwise comparisons

Univariate analyses with FDR correction, complemented by exploratory multivariate screening, identified several VOCs that differed statistically between groups in pairwise comparisons (Table 2). However, across all comparisons, substantial overlap in VOC distributions was observed, as illustrated by boxplots (Supplementary Figures 1–3 and Supplementary Results), indicating that no single compound provided clear separation at the individual level.

**TABLE 2:** Differential VOCs identified in three comparison groups and their characteristics.

VOC_ID	*P*-value	Fold change	VIP	AUC	Molecular name	Molecular formula
**Lung cancer vs. healthy participants**						
VOC@510.013	0.000956	1.136551	1.65696	0.757	Heptane	C_7_H_16_
VOC@529.907	0.02323	1.145522	2.12289	0.694	1-Methylthio-Propane	C_4_H_10_S
VOC@564.784	0.037502	0.619769	1.48415	0.589	6-Hydroxy-2-Hexanone	C_6_H_12_O_2_
VOC@668.997	0.007528	1.473895	1.243582	0.662	Styrene	C_8_H_8_
VOC@772.812	0.010957	0.663253	1.531225	0.664	o-Xylene	C_8_H_10_
**Pneumonia vs. healthy participants**						
VOC@495.218	0.000167	5.43221	1.34295	0.82	1,4-Pentadiene	C_5_H_8_
VOC@564.677	0.000006	3.488783	1.480356	0.823	Toluene	C_7_H_8_
VOC@604.254	0.001514	2.125165	1.238068	0.723	Butyl acetate	C_6_H_12_O_2_
VOC@637.426	0.001257	2.409148	1.213088	0.712	p-Xylene	C_8_H_10_
VOC@823.009	0.000775	2.94702	1.423677	0.706	D-Limonene	C_10_H_16_
VOC@862.358	0.000162	14.969418	1.430806	0.711	Iso-butyl nonyl carbonate	C_14_H_28_O_3_
**Lung cancer vs. pneumonia**						
VOC@474.085	0.008075	0.44858	1.011842	0.76	2-Methylbutane	C_5_H_12_
VOC@495.218	0.003852	0.334084	1.035689	0.766	1,4-Pentadiene	C_5_H_8_
VOC@564.677	0.000004	0.280476	1.494594	0.809	Toluene	C_7_H_8_
VOC@637.426	0.002517	0.420881	1.270734	0.737	p-Xylene	C_8_H_10_
VOC@772.889	0.0049	0.24997	1.166884	0.756	o-Xylene	C_8_H_10_
VOC@823.009	0.002798	0.39717	1.497614	0.691	α-Pinene	C_10_H_16_
VOC@862.358	0.000196	0.078595	1.40654	0.711	Iso-butyl nonyl carbonate	C_14_H_28_O_3_

VOC_ID, compound identification number; *P*-value, significance *P*-value for intergroup comparison; Fold Change, concentration change fold of disease group compared to control group (>1 indicates upregulation, <1 indicates downregulation); VIP, variable importance projection value (from OPLS-DA model); AUC, area under the curve when the compound is used as a single diagnostic marker. Screening criteria: *P* < 0.05 and VIP > 1.0.

When comparing lung cancer patients with healthy controls, a limited subset of VOCs showed statistically significant differences in abundance. Similarly, comparisons between pneumonia and healthy controls, as well as between lung cancer and pneumonia, revealed multiple VOCs with differential expression. These differences were modest in magnitude and consistently characterized by overlapping interquartile ranges across groups. Importantly, the observed VOC patterns reflected group-level shifts rather than distinct disease-specific signatures, consistent with the exploratory nature of the analysis.

Although statistically significant VOC differences were detected between groups, marked overlap in distributions underscores that these findings represent exploratory group-level patterns rather than definitive biomarker signals.

### Performance of machine learning models in binary classification

**FIGURE 1:**
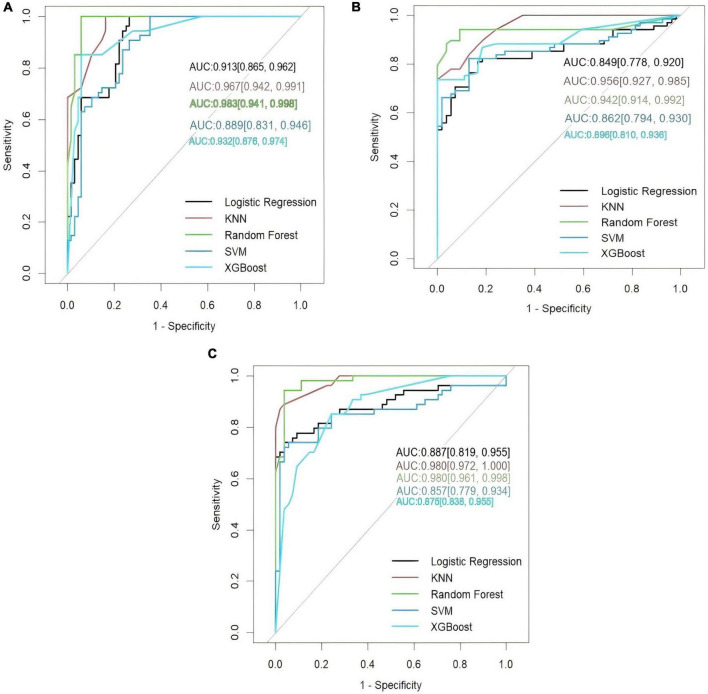
Receiver operating characteristic (ROC) curves of differences of VOCs. Based on the selected characteristic VOCs and baseline characteristics, this study constructed five machine learning models to distinguish between different groups. The effectiveness of these models was evaluated by generating receiver operating characteristic (ROC) curves and calculating the area under the ROC curve (AUC). **(A)** Lung cancer vs. healthy participants. **(B)** Pneumonia vs. healthy participants. **(C)** Lung cancer vs. pneumonia.

Machine learning analyses were conducted using pairwise binary classification frameworks to assess whether combinations of VOCs could improve group discrimination. Model performance metrics are summarized in Table 3 and Supplementary Table 1, with ROC curves shown in Figure 1.

**TABLE 3:** Comparison between training and test set AUC.

Model	Training_AUC	Testing_AUC
**Lung cancer vs. healthy volunteers**		
LR	0.801	0.887
KNN	0.97	0.98
RF	0.998	0.98
SVC	0.79	0.857
XGBoost	0.947	0.875
**Pneumonia vs. healthy volunteers**		
LR	0.88	0.849
KNN	0.976	0.956
RF	0.992	0.942
SVC	0.87	0.862
XGBoost	0.925	0.886
**Lung cancer vs. pneumonia**		
LR	0.933	0.913
KNN	0.980	0.967
RF	0.989	0.983
SVC	0.907	0.889
XGBoost	0.930	0.932

LR, logistic regression; KNN, k-nearest neighbors; RF, random forest; SVC, support vector machine; XGBoost, extreme gradient boosting. AUC, area under the receiver operating characteristic curve.

Across all pairwise comparisons, multivariate models achieved statistically robust discrimination, with AUC values consistently exceeding those expected by chance. While individual algorithms varied in performance, no single modeling approach uniformly outperformed others across all comparisons. Instead, model discrimination reflected the combined contribution of multiple VOC features, rather than reliance on any single compound.

Notably, despite favorable AUC values, overlap in predicted probabilities between groups was observed in all models, indicating limited separation at the individual subject level. These findings support the potential utility of VOC-based profiles for group discrimination in controlled settings, while emphasizing the current limitations for individual-level clinical classification.

Multivariate VOC-based models improved group-level discrimination compared with univariate analyses, but overlapping prediction distributions highlight the exploratory and hypothesis-generating nature of these results.

## Discussion

In this study, we conducted an exploratory investigation into the feasibility of using a portable VOC detection device combined with machine learning algorithms to perform pairwise discrimination among three groups: lung cancer, pneumonia, and healthy individuals, based on exhaled breath analysis. The results demonstrate that this approach can differentiate between lung cancer and healthy controls, as well as between pneumonia and healthy controls, in pairwise binary comparisons, with preliminary classification performance reflected in the AUC, sensitivity, specificity, and accuracy metrics. It should be emphasized that this was an internal validation study based exclusively on binary classification models; no multi-class modeling or external validation was performed at this stage. These findings suggest the potential utility of breath-based VOC analysis as a non-invasive exploratory tool for further investigation in clinical differentiation scenarios. However, its applicability as a clinical diagnostic tool requires further validation through independent cohorts, multi-class studies, and rigorous clinical trials.

This study shows that models constructed based on volatile organic compounds achieved high AUC values in the binary classification of lung cancer and pneumonia, demonstrating their discriminative ability in distinguishing these two conditions. However, a high AUC value does not necessarily mean it can be directly applied to clinical diagnostic scenarios. In real clinical diagnostics, decision-making often involves multiclassification, different disease prevalence rates, and different misclassification risks, thus requiring a more comprehensive and interpretable evaluation. While AUC provides a measure of overall discriminative ability, it cannot directly reveal the clinical distribution of classification errors. In real clinical environments, practicality is determined by the complete confusion matrix, which reveals the specific nature of classification errors. Special attention should be paid to false positives (e.g., pneumonia patients misclassified as lung cancer, leading to unnecessary invasive examinations) and false negatives (e.g., lung cancer patients misclassified as pneumonia, resulting in critical diagnostic delays). Additionally, post-test probabilities, particularly the positive predictive value (PPV), highly depend on the prevalence of the target disease in the intended population. Therefore, it is crucial to report the specificity, sensitivity, and trade-offs for each category in clinical contexts. Disease prevalence has a critical impact on the clinical utility of model predictions. In populations with a low prevalence of lung cancer, even if the model has high sensitivity and specificity, the positive predictive value may still be low, leading to a high false-positive rate and unnecessary further examinations and patient anxiety. Thus, when evaluating whether a model is suitable for the target scenario, the expected disease prevalence in that context must be analyzed.

A key methodological consideration in breathomics research is the influence of physiological and pathological confounders. To minimize potential bias, we carefully controlled for age, sex, smoking status, and comorbidities across study groups. This is particularly important because several VOCs, including isoprene, alkanes, and methylated alkanes, have been reported to correlate with age-related metabolic changes (8–10). In many previous studies, case groups were significantly older than controls, which may have confounded VOC-based discrimination. Similarly, smoking is a well-established risk factor for lung cancer and a major determinant of breath VOC composition. Matching smoking history between groups was therefore essential to reduce smoking-related bias. In addition, comorbid conditions unrelated to the target pulmonary diseases may independently alter VOC profiles; thus, harmonizing comorbidity distributions across groups was critical for identifying disease-associated VOC signatures rather than non-specific metabolic changes.

The present findings further highlight the advantages of integrating portable micro-GC-MS technology with machine learning models. Compared with conventional VOC analysis platforms such as GC-MS, PTR-MS, and electronic noses–which typically require complex infrastructure, high costs, and prolonged analysis times–the portable micro-GC system enables rapid, real-time VOC detection at the point of care. This portability and repeatability make the approach particularly attractive for longitudinal monitoring, disease screening, and potential deployment in resource-limited clinical environments.

Consistent with previous reports, the VOCs identified in lung cancer patients in this study predominantly belonged to the classes of alkanes, alkenes, ketones, and benzene derivatives (11). These compounds have been widely associated with altered lipid metabolism and oxidative stress in malignant tissues. From a mechanistic perspective, saturated and unsaturated hydrocarbons, such as heptane, 2-methylbutane, and 1,4-pentadiene, are thought to arise from lipid peroxidation of polyunsaturated fatty acids (PUFAs) within cell membrane phospholipids. Tumor cells are characterized by increased reactive oxygen species (ROS) production, which can induce oxidative damage to membrane lipids, proteins, and DNA. Enhanced lipid peroxidation may therefore plausibly contribute to elevated levels of volatile hydrocarbons detected in the exhaled breath of lung cancer patients. Likewise, ketone bodies and their derivatives may reflect altered mitochondrial β-oxidation of fatty acids, a metabolic pathway frequently upregulated in cancer cells (12). While these mechanisms provide a biologically plausible explanation, the present study was not designed to establish direct causal relationships between specific VOCs and underlying metabolic pathways.

In contrast, the pneumonia group exhibited a distinct VOC profile, with several compounds significantly elevated compared with healthy controls. Many of these VOCs have been previously associated with inflammatory responses and infectious processes. Aromatic hydrocarbons such as toluene and p-xylene have been reported to correlate with immune activation and inflammation (8, 13). Butyl acetate has been linked to acute lung injury and may reflect oxidative stress or membrane disruption during inflammatory responses (9). D-limonene, a terpenoid compound with reported anti-inflammatory properties, may also reflect host immune responses to pulmonary infection. Collectively, these findings suggest that inflammatory and infectious processes contribute to disease-specific VOC signatures in pneumonia, although the precise biological origins of individual compounds remain to be fully elucidated.

The ability of VOCs to distinguish lung cancer histological subtypes remains controversial. Previous studies have reported inconsistent findings, ranging from no detectable differences between subtypes (10), to statistically significant differences in selected VOCs (14), to subtype-dependent VOC profiles without clear statistical correlations (15). In the present study, subgroup analyses were limited by sample size, and no definitive conclusions regarding histological subtype discrimination could be drawn. These inconsistencies across studies underscore the need for larger, well-powered cohorts and standardized analytical pipelines to clarify the relationship between VOC profiles and tumor histology.

Volatile organic compounds detected in exhaled breath may originate from endogenous host metabolism, microbial metabolism, or host–pathogen interactions during infection or inflammation (16). In infectious lung diseases, invading microorganisms can generate a wide range of VOCs, including hydrocarbons, alcohols, ketones, and nitrogen- or sulfur-containing compounds. Previous studies have demonstrated pathogen-specific VOC patterns in pulmonary infections (17–21). In this study, seven VOCs were identified as discriminatory between pneumonia patients and healthy controls. Several of these compounds–such as pentadiene (22), toluene (23), p-xylene, limonene (24), and heptane–have been reported previously, whereas isobutyl nonyl carbonate was identified for the first time in this context. This compound is widely used as an industrial raw material and is not known to be synthesized endogenously, suggesting an exogenous origin (25). The most likely sources include disinfectants in hospital settings, volatiles from medical equipment, or personal care items for patients (Supplementary Table 2). Given that its source is independent of the underlying pathophysiological process, isobutylcarbonate should be considered a potential contaminant. We hypothesize that during pulmonary infection, increased metabolic activity and ROS production may alter the absorption, metabolism, or clearance of exogenous VOCs, leading to elevated detectable levels. This interpretation remains speculative and warrants further investigation.

Distinguishing early-stage lung cancer from pneumonia is clinically challenging because of overlapping symptoms and imaging features. When directly comparing lung cancer and pneumonia, the present study identified disease-specific differences in VOC profiles. Exhaled breath contains thousands of metabolites derived from host tissues, the respiratory tract, and associated microbiota. Both commensal and pathogenic microorganisms can produce VOCs across diverse chemical classes, some of which may serve as disease biomarkers (26). Aromatic hydrocarbons have been reported to exhibit high diagnostic value for pulmonary infections, including COVID-19 severity stratification (27), and are generally considered to be of exogenous origin (28). Our findings are consistent with these observations. In addition, fungal VOCs–such as terpenes including α-pinene and limonene–have been associated with pulmonary fungal infections (29, 30). The increased concentration of α-pinene observed in the pneumonia group in this study may therefore reflect microbial contributions to the breath VOC profile. This study aims to distinguish broad metabolic differences rather than VOCs targeting specific pathogens. Therefore, the next research is necessary to further analyze these VOC signals in a prospective cohort with clear etiological diagnosis, to identify which are the products of the host’s general inflammatory response to infection and which are direct markers of specific microbial metabolism. This will greatly enhance the specificity of VOC diagnostic tools, enabling them not only to distinguish lung cancer from pneumonia but also to further differentiate the cause of infection within pneumonia, thereby achieving more precise clinical decision-making.

Although several VOCs showed statistically significant differences between groups, these findings should be interpreted cautiously. Statistical significance alone does not guarantee clinical discriminative value. Substantial overlap in VOC distributions between groups was observed, indicating that individual VOCs are unlikely to function as reliable standalone biomarkers. This highlights the importance of multi-marker approaches and integrative modeling strategies. Accordingly, the present study emphasizes the use of multivariate machine learning models rather than reliance on single VOCs. The identified VOCs should be viewed as candidate features contributing to composite diagnostic models, whose performance and generalizability must be validated in independent cohorts.

There have been studies that have shown that certain alkanes in some lung cancer patients’ exhalation levels decrease, while aromatic compounds increase in lung inflammation. This is consistent with our research. The differences in the types of hydrocarbons and esters between pneumonia and lung cancer have also been proven in previous studies, supporting the idea that inflammation and tumor microenvironment may have different effects on specific VOC metabolism. However, the types of VOCs that were not found in previous studies may be due to differences in lung cancer classification, different pathogens, or variations in sample collection methods, analysis platforms, or statistical strategies. In this study, the lung cancer-specific VOC patterns may be more complex in the analysis of pneumonia confounding factors, and some overlapping compounds may show different changes in different controls. This can help optimize the selection of model features. However, further validation of the reproducibility of key compounds and exploration of combination biomarkers and standardized analysis workflows are still needed to improve the specificity of disease diagnosis.

Although the model constructed in this study showed high AUC in internal validation, we did not evaluate the calibration performance of the model. This means that the risk probability output of the model may not accurately reflect the true risk, especially when the prevalence of the target population is different from that of this study cohort. The future need to jointly validate and optimize the model’s discriminative and calibration performance in the external cohort.

Several limitations of this study should be acknowledged. First, the strict inclusion and exclusion criteria limit the generalizability of the findings. The results should be considered proof-of-concept and may not directly extend to patients with chronic respiratory diseases such as chronic obstructive pulmonary disease. Future studies should include more heterogeneous populations to assess model robustness in real-world settings. Second, although logistic regression was used for binary classification, other machine learning models capable of multi-class classification were also explored. The focus on binary differentiation between lung cancer and pneumonia reflects a clinically relevant diagnostic question; however, broader multi-disease classification frameworks warrant further investigation. Third, despite favorable model performance, the biological roles of individual VOCs in disease pathophysiology remain incompletely understood. Mechanistic studies are needed to clarify the sources and functional relevance of these compounds.

In conclusion, this study provides preliminary evidence that combining machine learning with exhaled VOC analysis offers a viable approach for the non-invasive differentiation of lung cancer and pneumonia from healthy controls. The methodological framework established here represents a foundational step toward addressing the clinical challenge of distinguishing these radiographically overlapping conditions. However, these findings are hypothesis-generating. Their potential to facilitate earlier diagnosis or improve clinical decision-making requires rigorous validation through multiclass modeling, external testing in independent cohorts, and prospective evaluation in real-world triage settings before any clinical application can be considered.

## Data Availability

The original contributions presented in this study are included in this article/Supplementary material, further inquiries can be directed to the corresponding author.
