# Correction: Exploratory analysis of exhaled volatile organic compounds for binary discrimination between lung cancer, pneumonia, and healthy controls using machine learning

**DOI:** 10.3389/fmed.2026.1857372

**Published:** 2026-05-20

**Authors:** Jing Wang, Haitian Li, Jianshen Yue, Yamei Song, Ning Wang, Wei Guo, Zhigang Cai

**Affiliations:** 1 The First Department of Pulmonary and Critical Care Medicine, The Second Hospital of Hebei Medical University, Shijiazhuang, China; 2 Department of Infectious Disease, The People's Hospital of Hengshui, Hengshui, China; 3 The First Department of Pulmonary and Critical Care Medicine, The People's Hospital of Cangzhou, Cangzhou, China; 4 Department of Pulmonary and Critical Care Medicine, The People's Hospital of Hengshui, Hengshui, China; 5 Hebei Key Laboratory of Respiratory Critical Care Medicine, Shijiazhuang, China; 6 Hebei Institute of Respiratory Diseases, Shijiazhuang, China

**Keywords:** exhaled breath analysis, exploratory study, lung cancer, machine learning, pneumonia, volatile organic compounds

Affiliation Hebei Key Laboratory of Respiratory Critical Care Medicine, Shijiazhuang, China; Hebei Institute of Respiratory Diseases, Shijiazhuang, China was omitted for author “Zhigang Cai”. This affiliation has now been added for author “Zhigang Cai”.

Affiliation “The First Department of Pulmonary and Critical Care Medicine, The Second Hospital of Hebei Medical University, Shijiazhuang, China” was omitted from the paper. This affiliation has now been added for author “Jing Wang”.

The correct affiliation and author listing appears below:

Jing Wang^1, 4^, Haitian Li^2^, Jianshen Yue^3^, Yamei Song^4^, Ning Wang^1, 4^, Wei Guo^4^, Zhigang Cai^1, 5, 6*^

1.The First Department of Pulmonary and Critical Care Medicine, The Second Hospital of Hebei Medical University, Shijiazhuang, China.

2.Department of Infectious Disease, The People's Hospital of Hengshui, Hengshui, China.

3.The First Department of Pulmonary and Critical Care Medicine, The People's Hospital of Cangzhou, Cangzhou, China.

4.Department of Pulmonary and Critical Care Medicine, The People's Hospital of Hengshui, Hengshui, China.

5.Hebei Key Laboratory of Respiratory Critical Care Medicine, Shijiazhuang, China.

6.Hebei Institute of Respiratory Diseases, Shijiazhuang, China.

The conflict of interest statement has been corrected to “The author(s) declared that this work was conducted in the absence of any commercial or financial relationships that could be construed as a potential conflict of interest.”

The original version of this article has been updated.

