# Supplementary material for: Exploratory analysis of exhaled volatile organic compounds for binary discrimination between lung cancer, pneumonia, and healthy controls using machine learning
Source: Front Med (Lausanne). 2026 Feb 23;13:1741424. doi: 10.3389/fmed.2026.1741424 (PMC12968303; doi:10.3389/fmed.2026.1741424)
Supplement: Supplementary file 1 [file Data_Sheet_1.docx]

**Supplementary Results**

**Differential VOC Profiles in Pairwise Comparisons**

Using univariate statistical testing with FDR correction and exploratory multivariate screening, several VOCs showed statistically significant differences in abundance between groups. Notably, despite statistical significance, substantial overlap in VOC distributions was observed across groups, as illustrated by the boxplots.

In the pairwise comparison between lung cancer patients and healthy controls, five VOCs demonstrated statistically significant differences. Compared with healthy controls, the lung cancer group exhibited lower peak areas of heptane (C₇H₁₆), propane, 1-(methylthio)- (C₄H₁₀S), and styrene (C₈H₈), while higher peak areas were observed for 2-hexanone, 6-hydroxy- (C₆H₁₂O₂) and o-xylene (C₈H₁₀).

Compared with healthy controls, the pneumonia group showed significantly higher peak areas for six VOCs, including 1,4-pentadiene (C₅H₈), toluene (C₇H₈), butyl acetate (C₆H₁₂O₂), p-xylene (C₈H₁₀), D-limonene (C₁₀H₁₆), and isobutyl nonyl carbonate (C₁₄H₂₈O₃).

In the comparison between lung cancer and pneumonia groups, seven VOCs displayed statistically significant differences. Specifically, peak areas of butane, 2-methyl-(C₅H₁₂), 1,4-pentadiene, toluene, p-xylene, o-xylene, α-pinene (C₁₀H₁₆), and isobutyl nonyl carbonate were lower in the lung cancer group than in the pneumonia group.

**Performance of Machine Learning Models in Binary Classification**

Machine learning analyses were conducted exclusively using pairwise binary classification frameworks. Five algorithms were evaluated using the VOC features identified in each comparison. Model performance metrics are summarized in Table 3, with receiver operating characteristic (ROC) curves shown in Figure 4. Comparisons between training and test set AUC values are presented in Table 4.

For lung cancer versus healthy controls, the random forest model achieved the highest AUC. While overall discrimination was strong at the group level, sensitivity and specificity varied across thresholds, indicating that model performance depended on operating point selection rather than uniform separation.

For pneumonia versus healthy controls, the k-nearest neighbors model yielded the highest AUC among the evaluated algorithms. This performance reflected the collective contribution of multiple VOC features rather than dominance of any single compound.

In the comparison between lung cancer and pneumonia, the random forest model again demonstrated the highest AUC. However, despite favorable summary metrics, overlap in predicted probabilities was observed.

Across all comparisons, machine learning results indicate that VOC-based models can achieve statistically robust discrimination in binary settings, but do not imply definitive clinical classification at the individual level.

**Supplementary Figure 1. Box plots of differences of VOCs between lung cancer patients and healthy participants**

Compare the area under the curve of different VOC peak levels between healthy participants and lung cancer patients. In the box plot, the box represents the 25^th^-75^th^ percentiles (interquartile range, IQR), the line inside the box is the median, and points beyond the whiskers are defined as outliers. The difference between the two groups was statistically analyzed using the Mann-Whitney U test, and the P values obtained from the test have been corrected for multiple testing using the FDR method to control the risk of false positives caused by multiple comparisons.Data is expressed in terms of integrated peak area, and overlapping peaks are resolved by fitting to a Gaussian mixture model, with the integrated peak area of each resolved compound calculated as a quantitative indicator.


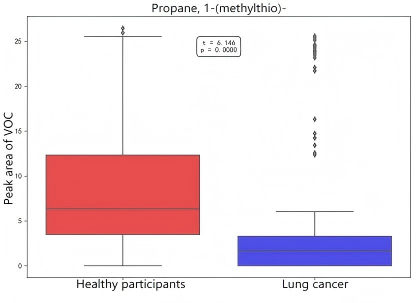

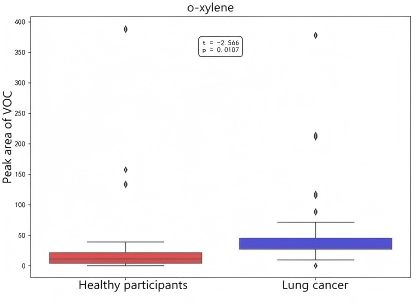


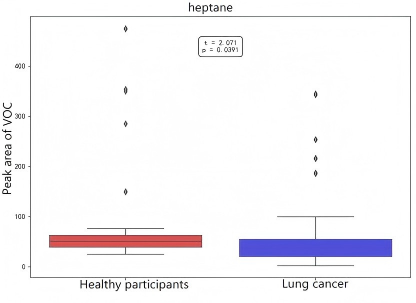

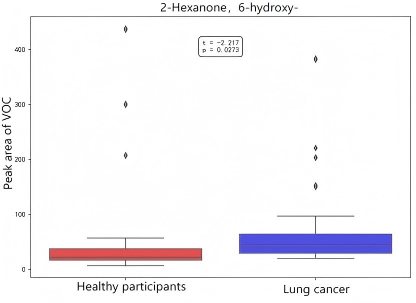


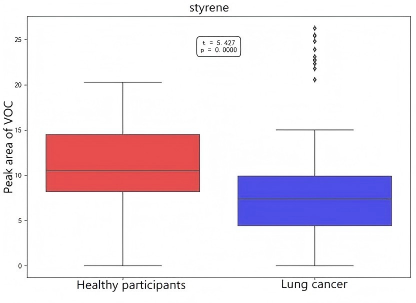


**Supplementary Figure 2. Box plots of differences of VOCs between pneumonia patients and healthy participants**

Compare the area under the curve of different VOC peak levels between healthy participants and pneumonia. In the box plot, the box represents the 25th–75th percentiles (interquartile range, IQR), the line inside the box indicates the median, and points beyond the whiskers are defined as outliers. The difference between the two groups was statistically analyzed using the Mann-Whitney U test, and the P values obtained from the test have been corrected for multiple testing using the FDR method to control the risk of false positives due to multiple comparisons.Data is expressed in terms of integrated peak area, and overlapping peaks are resolved by fitting to a Gaussian mixture model, with the integrated peak area of each resolved compound calculated as a quantitative indicator.


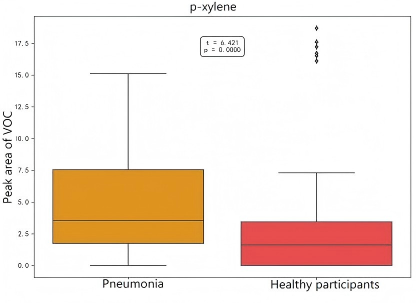

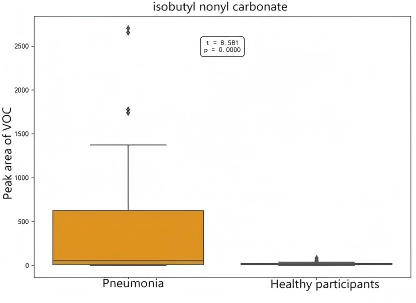


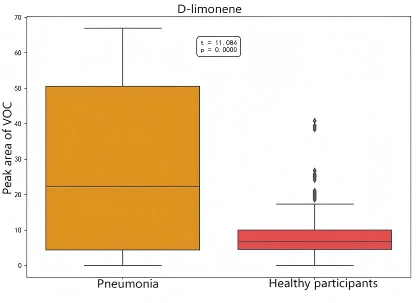

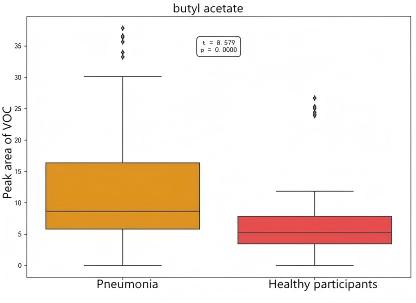


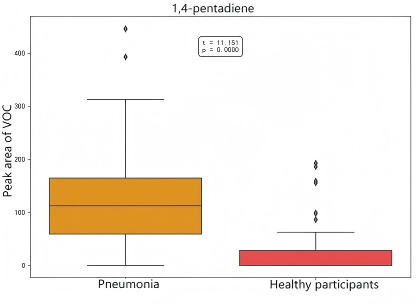

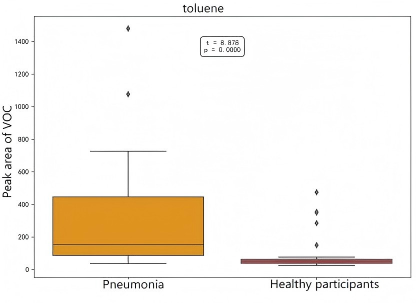


**Supplementary Figure 3. Box plots of differences of VOCs between lung cancer patients and pneumonia patients**

Compare the area under the curve of different VOC peak levels between Lung cancer and pneumonia. In the box plot, the box represents the 25th–75th percentiles (interquartile range, IQR), the line inside the box indicates the median, and points beyond the whiskers are defined as outliers. The difference between the two groups was statistically analyzed using the Mann-Whitney U test, and the P values obtained from the test have been corrected for multiple testing using the FDR method to control the risk of false positives due to multiple comparisons.Data is expressed in terms of integrated peak area, and overlapping peaks are resolved by fitting to a Gaussian mixture model, with the integrated peak area of each resolved compound calculated as a quantitative indicator.


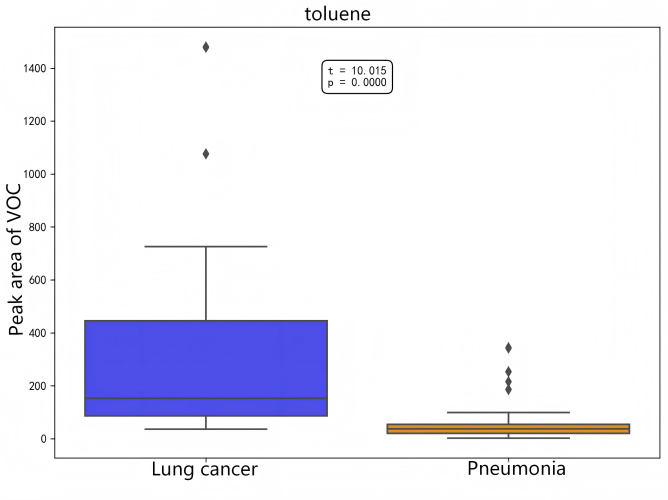

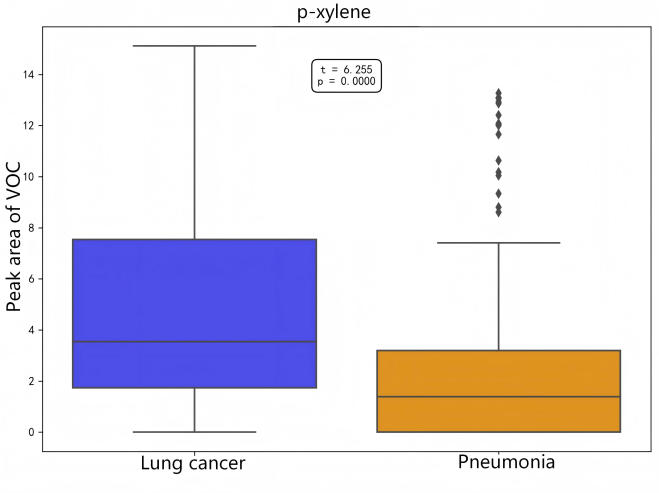

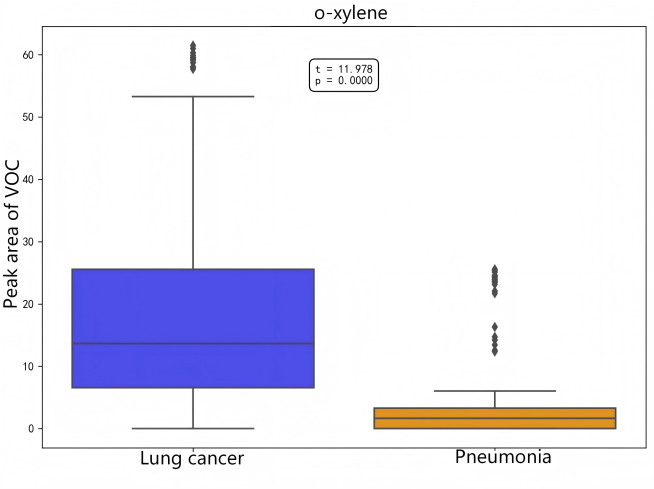

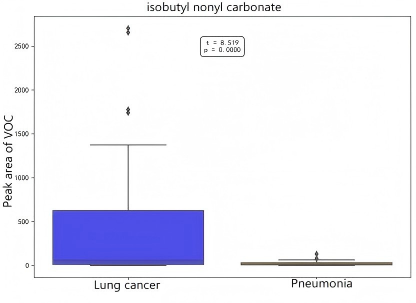

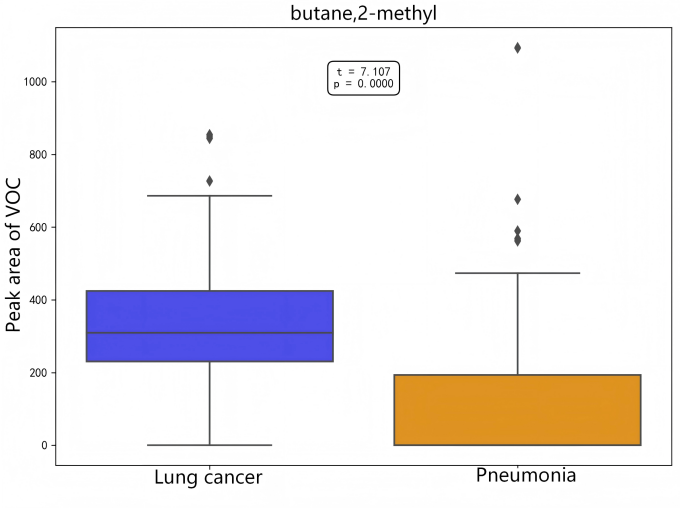

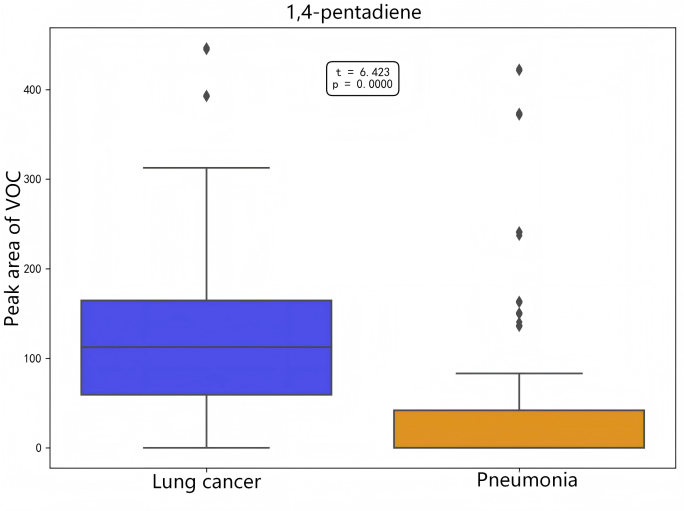

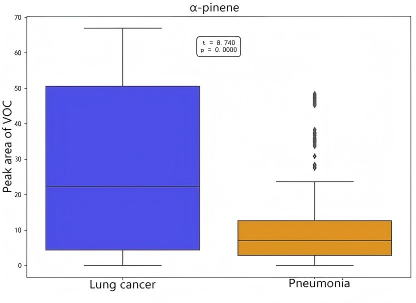


**Supplementary Table 1. Model performance analysis based on characteristic volatile organic compounds (VOCs)**

|  | **AUC** | **95% CI** | **F1** | **Accuracy** | **Sensitivity** | **Specificity** | **PPV** | **NPV** |
| --- | --- | --- | --- | --- | --- | --- | --- | --- |
| **Lung cancer vs. healthy participants** | | | | | | | | |
| LR | 0.887 | [0.819, 0.955] | 0.796 | 0.810 | 0.851 | 0.759 | 0.780 | 0.837 |
| SVC | 0.857 | [0.779, 0.934] | 0.760 | 0.778 | 0.852 | 0.704 | 0.742 | 0.826 |
| RF | 0.980 | [0.972, 1.000] | 0.954 | 0.954 | 0.944 | 0.963 | 0.963 | 0.945 |
| KNN | 0.980 | [0.961, 0.998] | 0.857 | 0.870 | 0.963 | 0.778 | 0.813 | 0.955 |
| XGBoost | 0.875 | [0.838, 0.955] | 0.796 | 0.806 | 0.852 | 0.759 | 0.780 | 0.837 |
| **Pneumonia vs. healthy participants** | | | | | | | | |
| LR | 0.849 | [0.778, 0.920] | 0.803 | 0.811 | 0.765 | 0.870 | 0.881 | 0.746 |
| SVC | 0.862 | [0.794, 0.930] | 0.797 | 0.787 | 0.662 | 0.944 | 0.938 | 0.689 |
| RF | 0.942 | [0.914, 0.992] | 0.901 | 0.910 | 0.897 | 0.926 | 0.938 | 0.877 |
| KNN | 0.956 | [0.927, 0.985] | 0.852 | 0.861 | 0.824 | 0.907 | 0.918 | 0.803 |
| XGBoost | 0.896 | [0.810, 0.936] | 0.796 | 0.811 | 0.794 | 0.833 | 0.857 | 0.763 |
| **Lung cancer vs. pneumonia** | | | | | | | | |
| LR | 0.913 | [0.865, 0.962] | 0.809 | 0.795 | 0.815 | 0.779 | 0.746 | 0.841 |
| SVC | 0.889 | [0.831, 0.946] | 0.797 | 0.770 | 0.722 | 0.809 | 0.750 | 0.786 |
| RF | 0.983 | [0.941, 0.998] | 0.948 | 0.943 | 0.944 | 0.941 | 0.927 | 0.955 |
| KNN | 0.967 | [0.942, 0.991] | 0.902 | 0.893 | 0.907 | 0.882 | 0.860 | 0.923 |
| XGBoost | 0.932 | [0.876, 0.974] | 0.874 | 0.861 | 0.852 | 0.868 | 0.836 | 0.881 |

LR: Logistic Regression; KNN: K-Nearest Neighbors; RF: Random Forest; SVC: Support Vector Machine. AUC: Area Under the Receiver Operating Characteristic Curve. All models were evaluated on the same training set and an independent test set

# Supplementary Table 2. Potential biomarkers of VOCs in respiratory diseases and source analysis

| **Compound** | **Molecular formula** | **Potential endogenous sources/pathophysiological associations** | **Common exogenous/environmental exposure sources** |
| --- | --- | --- | --- |
| Acetic acid, butyl ester | C6H12O2 | Previous studies have associated it with acute lung injury, potentially reflecting oxidative stress or cell membrane damage during the inflammatory response. | It is widely used as a solvent for paints, coatings, fragrances and fragrances; Also used as a food additive. |
| Butane, 2-methyl- | C5H12 | As a short-chain alkane, it may be produced by the lipid peroxidation of polyunsaturated fatty acids, is associated with oxidative stress, and has been reported to change in respiratory phenomics studies of lung diseases such as chronic obstructive pulmonary disease. |  |
| D-Limonene | C10H16 | A terpene compound with anti-inflammatory properties. The level changes in patients with pneumonia may reflect the immune response of the host to the lung infection. | The main components of essential oils extracted from citrus fruit peels; used in food, fragrance, and cleaning agents. |
| Heptane | C7H16 | Considered to be a product of lipid peroxidation. Increased tumor-related ROS and enhanced lipid peroxidation may lead to changes in its levels in exhaled breath of lung cancer patients. |  |
| 2-Hexanone, 6-hydroxy- | C6H12O2 | It belongs to the ketone group. Ketone bodies and their derivatives may reflect changes in the mitochondrial β-oxidation process of fatty acids, a pathway that is often upregulated in cancer cells. |  |
| Isobutyl nonyl carbonate | C14H28O3 | The human body lacks known endogenous synthetic pathways. This study first identified the compound in the context of pneumonia, suggesting it may serve as an exogenous exposure marker, with its concentration changes during infection potentially related to altered host metabolic clearance. | It may come from additives in some personal care products, cosmetics, and possible sources include disinfectants in hospital environments, volatile substances in medical devices, or the personal care products of patients. |
| Propane, 1-(methylthio)- | C4H10S | It may be a derivative of sulfur-containing amino acid metabolism (such as methionine), and its changes may be related to metabolic changes under specific pathological conditions. | It may be present in some fermented foods |
| α-Pinene | C10H16 | A terpene compound. Its concentration increased in the pneumonia group, possibly reflecting the impact of microbial metabolic activity in lung infections (including fungal infections) on the exhaled breath profile. | It is common in some spices, cleaning products, and air fresheners. |
| 1,4-Pentadiene | C5H8 | It is a product of peroxida formation of polyunsaturated fatty acids (PUFA) lipids in cell membranes. The level may be increased by the increased level of reactive oxygen species (ROS) in tumor microenvironment. |  |
| Styrene | C8H8 | Endogenous production is limited, and is often considered a potential confounding factor in respiratory group studies that requires caution. | It is the main raw material of polystyrene, synthetic rubber, resin; it is also common in cigarette smoke and automobile exhaust. |
| Toluene | C7H8 | Previous studies have reported associations with immune activation and inflammatory processes, which may reflect host metabolic changes or microbial activity under infection or inflammatory conditions. | Common components in paint, coatings, adhesives, nail polish, and cigarette smoke. |
| o-Xylene | C8H10 | It belongs to the aromatic hydrocarbons, and its pattern of change may reflect disease-specific metabolic activity, but the specific endogenous pathway is not clear. | Common components of paint, printing inks, pesticides, and automotive exhaust. |
| p-Xylene | C8H10 | Its level changes are often associated with inflammatory responses and may increase in exhaled breath of patients with infectious diseases such as pneumonia. |  |
